# Physicochemical Characterizations and Pharmacokinetic Evaluation of Pentazocine Solid Lipid Nanoparticles against Inflammatory Pain Model

**DOI:** 10.3390/pharmaceutics14020409

**Published:** 2022-02-14

**Authors:** Zaheer Ullah Khan, Anam Razzaq, Ahsan Khan, Naeem Ur Rehman, Hira Khan, Taous Khan, Ashraf Ullah Khan, Norah A. Althobaiti, Farid Menaa, Haroon Iqbal, Naveed Ullah Khan

**Affiliations:** 1 Department of Pharmacy, Abbottabad Campus, COMSATS University Islamabad, Abbottabad 22060, Pakistan; zaheerkhan@ciit.edu.pk (Z.U.K.); Fa15-r60-001@cuiatd.edu.pk (A.K.); naeem@gu.edu.pk (N.U.R.); taouskhan@cuiatd.edu.pk (T.K.); 2 Department of Pharmaceutics, College of Pharmaceutical Sciences, Soochow University, Suzhou 215123, China; anamrazzaq.ajk@gmail.com; 3 Division of Pharmaceutics and Pharmacology, College of Pharmacy, Ohio State University, Colombus, OH 43210, USA; khan.115@osu.edu; 4 Department of Pharmacy, Quaid-i-Azam University, Islamabad 45320, Pakistan; aukhan@bs.qau.edu.pk; 5 Faculty of Pharmaceutical Sciences, Abasyn University, Peshawar 25000, Pakistan; 6 Department of Biology, College of Science and Humanities-Al Quwaiiyah, Shaqra University, Al Quwaiiyah 19257, Saudi Arabia; nalthobaiti@su.edu.sa; 7 Department of Oncology and Nanomedicine, California Innovations Corporation, San Diego, CA 92037, USA; 8 Institute of Basic Medicine and Cancer (IBMC), Chinese Academy of Sciences (CAS), The Cancer Hospital of the University of Chinese Academy of Sciences (Zhejiang Cancer Hospital), Hangzhou 310022, China; harooniqbal415@hotmail.com; 9 Department of Pharmacy, Gujrat Campus, University of Lahore, Lahore 50700, Pakistan

**Keywords:** pentazocine, solid lipid nanoparticles, oral bioavailability, first-pass metabolism, inflammation, controlled and sustained drug release

## Abstract

Pentazocine (PTZ), a narcotic-antagonist analgesic, has been extensively used in the treatment of initial carcinogenic or postoperative pain. Hepatic first-pass metabolism results in low oral bioavailability and high dose wastage. Herein, 10 mg (-)-Pentazocine (HPLC-grade) was incorporated to solid lipid nanoparticles (SLNs) using a double water-oil-water (*w*/*o*/*w*) emulsion by solvent emulsification–evaporation technique, followed by high shear homogenization to augment its oral bioavailability, considering the lymphatic uptake. The resulting SLNs were characterized for zeta potential (ZP), particle size (PS), and polydispersity index (PDI) using a zetasizer. The entrapment efficiency (EE) and loading capacity (LC) were calculated. Chemical interactions, through the identification of active functional groups, were assessed by Fourier-transformed infrared (FTIR) spectroscopy. The nature (crystallinity) of the SLNs was determined by X-ray diffractometry (XRD). The surface morphology was depicted by transmission electron microscopy (TEM). In vitro (in Caco-2 cells) and in vivo (in male Wistar rats) investigations were carried out to evaluate the PTZ release behavior and stability, as well as the cellular permeation, cytotoxicity, systemic pharmacokinetics, antinociceptive, anti-inflammatory, and antioxidative activities of PTZ-loaded SLNs, mainly compared to free PTZ (marketed conventional dosage form). The optimized PTZ-loaded SLN2 showed significantly higher in vitro cellular permeation and negligible cytotoxicity. The in vivo bioavailability and pharmacokinetics parameters (t_1/2_, Cmax) of the PTZ-loaded SLNs were also significantly improved, and the nociception and inflammation, following carrageenan-induced inflammatory pain, were markedly reduced. Concordantly, PTZ-loaded SLNs showed drastic reduction in the oxidative stress (e.g., malonaldehyde (MDA)) and proinflammatory cytokines (e.g., Interleukin (IL)-1β, -6, and TNF-α). The histological features of the paw tissue following, carrageenan-induced inflammation, were significantly improved. Taken together, the results demonstrated that PTZ-loaded SLNs can improve the bioavailability of PTZ by bypassing the hepatic metabolism via the lymphatic uptake, for controlled and sustained drug delivery.

## 1. Introduction

SLNs are spherical particles of nanometer range, immersed in water or aqueous surfactant solution, using either lipophilic or hydrophilic drugs [1]. SLNs, generally termed as “nano safe” carriers, elicit incredibly low acute and chronic toxicity, while improving the bioavailability and stability of poor water-soluble molecules, making them utmost evolving nanotools, with several applications in different fields, such as drug delivery and clinical medicine [2,3]. Indeed, SLNs represent highly stable, safe, and biodegradable colloidal nanocarriers that can be modified to exhibit various advantages, compared to liposomes and polymeric nanoparticles (NPs) [4]. SLNs display exceptional tolerability, as they are made up from the lipids and carriers and have encouraging propensity for drug development and evaluation [5]. Drug-targeted delivery and enhanced bioavailability are major parameters for minimizing drug-induced deleterious side effects [6]. Because different fabricated NPs and drugs displayed low solubility and poor pharmacokinetic profiles, SLNs appeared as ideal delivery systems and have attracted increasing attention during recent years [7]. The oral absorption of SLNs-encapsulated drugs have been shown to be enhanced via paracellular transport and Peyer’s patches, also called aggregated lymphoid nodules [8]. Lymphatic transport from the intestine provides alternate way to bypass the pre-systemic liver metabolism via thoracic lymph vessel, which combines with the circulation system at the junction point of the jugular and left subclavian veins [9].

PTZ (IUPAC name: (1*R*,9*R*,13*S*)-1,13-dimethyl-10-(3-methylbut-2-enyl)-10-azatricyclo [7.3.1.02,7]trideca-2(7),3,5-trien-4-ol) is a benzomorphan derivative (Figure S1), an opioid painkiller employed for moderate to severe analgesia, often needed and used in clinics [10]. It is indicated to manage severe pain (e.g., acute renal, biliary colic, cancer pain, menstrual pain, and postoperative) [11]. The main shortcomings, associated with PTZ, are its short half-life (t_1/2_ = 1–2 h) and limited oral bioavailability (<20%), due to protracted hepatic breakdown and low solubility, subsequently entailing various doses to execute the steady-state concentration and pain relief [12,13].

In this context, we hypothesized that the encapsulation of PTZ into SLNs could enhance the bioavailability of PTZ prone to first-pass metabolism and may represent a promising drug delivery system (DDS) for oral administration and lymphatic uptake. Therefore, we evaluated, both in vitro and in vivo, an optimized PTZ-loaded SLNs delivery nanosystem to enhance oral administration and systemic availability of PTZ, by avoiding liver metabolism and toxicity. To the best of our knowledge, this is a pioneered study to evaluate the PTZ-loaded SLNs for systemic availability enhancement by bypassing the hepatic metabolism via the lymphatic route, for controlled and sustained drug delivery.

## 2. Materials and Methods

### 2.1. Chemical and Reagents

The (-)-PTZ was gifted by Global Pharmaceutical Industry, Islamabad, Pakistan. The polysorbate 20 and 80 were obtained from Sigma-Aldrich (Burlington, MA, USA). Soya lecithin was purchased from Lipoid GmbH (Ludwigshaften, Germany). Sucrose, cetyl palmitate, and stearic acid were purchased from Cognis GmbH (Mannheim, Germany). Glycerol monostearate (GMS) and cetyl-alcohol were obtained from Acros Organics (Fair Lawn, New Jersey, USA). Dichloromethane (DCM), acetone, and acetonitrile (ACN), of analytical grade, were purchased from Merck (Darmstadt, Germany). Distilled water (dH_2_O) was obtained from the Pharmacy Department, COMSATS University Islamabad, Abbottabad campus, Pakistan.

### 2.2. Formulation of Nanocarriers

#### 2.2.1. Screening of Solid Lipids

Solid lipids were selected on behalf of drug solubility in molten lipid [14]. A total of 5 mg of PTZ was added to observe the solubility in molten lipids (stearic acid, cetyl alcohol, cetyl palmitate, and glycerol monostearate). The amount of molten lipids required to solubilize the active pharmaceutical ingredient (API) was noted with the naked eye, up to 24 h. The final step was the formation of clear solution of molted lipid and API.

#### 2.2.2. Preparation of SLNs

To formulate SLNs, a double emulsion (*w*/*o*/*w*) solvent emulsification–evaporation (DESE) technique, followed by high shear homogenization, using homogenizer T25 IKA Ultra-Turrax^®^ (Sigma-Aldrich Chemie GmbH, Taufkirchen, Germany), was used, following the protocol published by Varshosaz et al. (2010), with slightly modifications [15]. PTZ (10 mg), soya lecithin, and lipids (cetyl alcohol/stearic acid (80/70 mg)) were liquified in organic solvent mixture (DCM:acetone). The aqueous phase was added dropwise to the lipid phase with sonication to prepare *w*/*o* nanoemulsion. The nanoemulsion was subsequently added to dH_2_O, holding 1.6% emulsifier, utilized in first step while stirring (1300 rpm). Then, the system was homogenized at 15,000 rpm and stirred overnight to evaporate organic solvent or mixture of solvents. The SLNs were obtained in dry form after lyophilization using a freeze dryer (Model Cryodos-50, 2008, TELSTAR, Terrassa, Spain). The water contents were subsequently removed from the product at −35 °C by decreasing pressure, and sucrose (1% *w*/*w*) was added to the formulation to inhibit particle increase.

#### 2.2.3. Optimization of SLNs Formulations

Design of expert (DoE) was selected as a statistical tool for optimizing the formulations and studying the effectiveness of formulation ingredients (independent variables) on ZP, PS, PDI, EE, and LC (dependent variables) [15]. Every factor of design was checked at two levels (high and low). Using Stat-Ease Design-Expert (10.0.3), by Taghuchi L8 model, eight formulations (Table 1) were prepared. The lecithin and lipids ratios, lipid types, surfactants type, their concentrations, DCM, acetone ratio, homogenizer speed, and time were the independent variables (Table 1).

#### 2.2.4. High-Performance Liquid Chromatography (HPLC)

For quantification of PTZ, the HPLC (model Waters^®^ e2695; Milford, MA, USA), attached with photo diode array (PDA) detector, model 2998 (Waters, Milford, MA, USA), was used. The C18 (L. 150 mm × I.D. 4.6 mm, 5 µm PS, 100 Å pore size) column was employed for L. chromatographic separation using a validated method, with slight modifications [16]. The mobile phase was comprised of acetonitrile (ACN) and H_2_O, in a 90:10 ratio. The injection volume of 20 µL was used at the 0.4 mL/min flow rate. The ultraviolet (UV) detection was carried out at 278 nm. PTZ was also characterized as a pure compound (Figure S2).

### 2.3. Physicochemical Characterizations

#### 2.3.1. Micromeritics and Surface Charge

The PS (diameter size), ZP (electrokinetic potential), and PDI (homogeneity) of the colloidal formulations were all determined by Zetasizer Nano ZS (Malvern, UK), founded on photon correlation spectroscopy at a fixed angle 90°. SLNs were diluted 20 folds in dH_2_O for ZP analysis [17].

#### 2.3.2. Entrapment Efficiency and Drug Loading Capacity

The drug contents were measured by HPLC at 278 nm by dissolving SLNs into ACN, as described herein previously. A total of 2 mg of PTZ-SLNs were liquified into 1 mL of can and vortexed and tailed by centrifugation at 12,500 rpm (Centrifuge 5810; Eppendorf, Hamburg, Germany) for 10 min to obtain a clear supernatant. Then, the supernatant was sieved by 0.45 μm syringe filter and analyzed by HPLC [16,18]. EE (%) and LC (%) were calculated by the following equations:

(1) $$\mathrm{EE}\;\left(\%\right)=\frac{Ws}{Wt}\times 100Ws=Wt-Wf$$

(2) $$\mathrm{LC}\;\left(\%\right)=\frac{Ws}{Wlipid}\times 100$$

where Wf = free drug in supernatant (free non-entrapped drug); Ws = amount of drug loaded in the SLNs; Wt = total amount of drug added in the formulation; and Wlipid = total amount of lipid vehicle.

### 2.4. FTIR and XRD Analyses

FTIR was performed to get information on the chemical interactions/reactions taking place between PTZ and the excipient (SLN). The IR spectra of drug, surfactant, co-surfactant, and loaded and unloaded composite spheres were recorded in the range of 400 to 4000 cm^−1^ using KBr pellet via FTIR spectroscopic instrument (Spectrum 400; Perkin Elmer, Waltham, MA, USA) [18].

The crystallinity was studied by XRD analysis of free PTZ, PTZ-loaded SLNs, and unloaded SLNs. The diffractometer range, which was employed at *b*/*w* 10° to 80°, with 2θ angle of diffraction at 0.05°/min, 45 kV voltages, and 20 mA current [19].

### 2.5. Morphological Attributes

SLNs were evaluated for morphology by TEM, using the JEOL-JEM1010 instrument (JEOL Ltd., Tokyo, Japan). A diluted PTZ-SLNs solution was dropped on a 400-mesh, carbon-coated, copper grid, in order to let the surface adsorption tailed by 1% phosphor-tungstic acid aqueous solution -ve staining. Then, the samples were dried at ambient/room temperature (RT) and imaged by TEM, set up at the accelerating voltage of 200 kV [19].

### 2.6. In Vitro Drug Release

The drug release was studied in 1× simulated gastric fluid (SGS) and 1× phosphate-buffer saline (PBS), by using the dialysis-bag method [20], at pH 1.2 (SGS) for 2 h and at pH 6.8 (PBS) for 12 h 24 h. The bags were saturated in double-distilled water (ddH2O). 2 mg of PTZ-SLN, in 1 mL PBS, was placed in the dialysis bag and kept at 37 ± 1 °C under magnetic stirring at 100 rpm in 10 mL tube containing SGS or PBS [9]. At regular time intervals, aliquots of dialysate samples were withdrawn, and replenished by same volume of fresh medium. The samples were withdrawn at various time points (i.e., 0, 10, 20, and 30 min, as well as 1, 2, 3, 4, 6, 7, 8, 9, 10, and 12 h) for kinetics analysis, filtered by a 0.2 μm filter, and analyzed by HPLC at 278 nm. All the samples were carried out in triplicates, independently.

### 2.7. Stability Studies

The stability studies were fulfilled for optimized lyophilized SLNs in transparent sealed test tubes and stored in the dark area at ~25 ± 0.5 °C. Stability was investigated in storage period for three months. SLNs (PTZ-loaded) were checked for stability by measuring their PS, PDI, and ZP at day 0, 1, 7, 14, 30, 60, and 90 [17].

### 2.8. Culture of Caco-2 Cells and Caco-2 Cells-Based Assays

Caco-2 cells were received from CHI Scientific Ltd. (Wuxi, China). Caco-2 is an immortalized cell line of human colorectal adenocarcinoma cells, which has been widely used as a model of the intestinal epithelial barrier. Cells were cultured in DMEM supplemented with 20% fetal bovine serum (FBS0) and antibiotics (100 U/mL penicillin and 100 U/mL streptomycin). The cells were then incubated in a humidified incubator at 37 °C under 5% CO_2_ [20,21].

#### 2.8.1. In Vitro Caco-2 Cytotoxicity Assay

Caco-2 cells were seeded into 96-wells plates at density of 1 × 10^3^/well. After the cell morphology became normal and the confluence of the cells reached more than 80%, PTZ-loaded SLNs were added at a range of concentrations (0–100 µg/mL) and incubated for 48 to 72 h. 40 µL of MTT reagent in PBS (5 mg/mL) was then added and incubated for 4 h. The medium was eventually removed and 150 µL DMSO was added and shake for 15 min at 100 rpm. Then OD values were analyzed by spectrophotometry using a microplate reader (BMG Labtech, Ortenberg, Germany) at 570 nm and the cell viability was calculated as follow [20]:

$$\mathrm{Cell}\;\mathrm{viability}\;\left(\%\right)=\frac{\mathrm{OD}\;\mathrm{value}\;\mathrm{of}\;\mathrm{dosing}\;\mathrm{group}-\mathrm{blank}}{\mathrm{OD}\;\mathrm{value}\;\mathrm{of}\;\mathrm{control}\;\mathrm{group}-\mathrm{blank}}\times 100$$

where, OD means optical density; dosing/testing group represents PTZ-loaded SLNs; control group represents free PTZ; and blank represent PBS group.

#### 2.8.2. Cellular Permeability Studies

Caco-2 cells were seeded into a 24-wells transwell insert (3.0 μm pore size, Millipore, MA, USA) at a density of 1 × 10^5^/cm^2^. After 17–21 days, the transepithelial electrical resistance (TEER) was measured by an EVOM volt ohmmeter (World Precision Instruments, Sarasota, FL, USA) to estimate the integrity of cell monolayer at TEER values of 150 to 300 Ω·cm^2^. Cells were washed thrice, and PTZ-loaded SLNs were added at dose of 5 μg in 200 µL Hanks’ Balanced Salt Solution (HBSS) to the upper chamber of the transwell while 1.0 mL HBBS was added to the lower chamber. Cells were subsequently maintained at 37 °C with orbital shaking at 50–60 rpm/min. At various pre-determined intervals (1, 2, 3, 6 h), 0.5 mL HBSS was collected, and an equal volume of fresh HBSS was promptly added to the lower chamber. The collected samples were freeze-dried and re-dissolved in ACN. The PTZ concentration was measured by HPLC as previously mentioned. The cumulative transport mass (Q, μg) was calculated using the following equation [11]:

Q=Ci ×V+∑Ci−1×0.5

where, Q represents the cumulative transport mass (μg); V is the volume of the solution in lower chamber of transwell (mL); and Ci stands for the PTZ concentration (μg/mL).

### 2.9. In Vivo Studies

#### 2.9.1. Animal Care

Animals were kept under the “standard conditions” in the animal house of pharmacy department, COMSATS University Islamabad (CUI), Abbottabad campus, Pakistan. Male Wistar rats (N = 32), purchased from NIH, Islamabad, Pakistan, weighing 250 ± 20 g, were employed for the present study. A total of 12 rats (2 groups, n = 6) were used for the pharmacokinetic studies, while the remaining 20 rats (4 groups, n = 5) were used in antinociceptive and anti-inflammatory activities. All animal studies were complied with the requirements of National Act on the dealing with experimental animals (COMSATS University Islamabad, Abbottabad campus, Pakistan). At ambient temperature/RT (25–30 °C), animals were restrained in animal house with relative humidity (RH) 45–55% and 12 h light-dark cycle. Animals were fed a pellet diet and water (Aqua Guard pure water) ad libitum. The rats were kept hungry up to 12 h but had free access to water. Experimental rats (5/6 per cage) were housed during acclimatization and treatment. The stainless-steel top grill cages (H × W × l of 1290 cm × 220 mm × 140 mm) had the facilities for rodent’s food and water *ad libitum*. The study was conducted according to the university animals’ ethics committee guidelines [8] and approved by the ethical review board (ERB), under # CIIT1678-2020.

#### 2.9.2. Pharmacokinetic Studies

The rats were classified in two groups, each of 6 animals (n = 6/group), following a published approach of pharmacokinetic studies [22,23]. Briefly, through oral gavage, the first group (control group) received 5 mg per kg of body weight of the free marketed PTZ solution [23], while the second group (test group) was administered with the PTZ-loaded SLNs suspension, containing the same amount of PTZ (5 mg/kg).

Blood samples (300 μL) were withdrawn at various time points during 6 h (i.e., 0, 10, 30, 60, 120, 180, 240, 360, and 480 min) via heart puncturing and collected in EDTA tubes. Collected blood was centrifuged for 10 min at 3500 rpm. The obtained plasma was stored at −20 °C for further analyses. Then, the precipitation of plasma protein was performed by mixing ACN and plasma, which mixture was vortexed for 2 min and centrifuged at 4000 rpm for 5 min. Eventually, the supernatant was filtered via 0.22 μm syringe filter and analyzed through HPLC to calculate the pharmacokinetic parameters, such as biological half-life (t1/2) and area-under the concentration curve (AUC) [8].

#### 2.9.3. Antinociceptive and Anti-Inflammatory Activities

In the present study, the animals (rats) were randomly assigned to four groups (20 rats, n = 5), i.e., vehicle control (received only orally 1×PBS, Group 1), carrageenan (received 200 μL/paw of the 1% *w*/*v* solution, Group 2), free PTZ (received intraplantar (i.pl) administration of carrageenan 200 μL/paw of the 1% *w*/*v* solution + free PTZ, orally administrated at the dose of 5 mg/kg, Group 3), and PTZ-loaded SLN2 (received i.pl administration of carrageenan 200 μL/paw of the 1% *w*/*v* solution + PTZ-loaded SLN2, orally administrated at the dose of 5 mg/kg, Group 4). The carrageenan was administered into Groups 3 and 4, 30 min before the treatment with free PTZ and PTZ-loaded SLN2, respectively.

##### Evolution of Paw Edema

The carrageenan-induced inflammation and nociception is a well-established model to evaluate pre-clinically anti-inflammatory and analgesic activities of drugs. The single i.pl administration of carrageenan (200 μL/paw) triggers the inflammation by inducing the release of the inflammatory cytokines and oxidative stress [24]. The paw edema was measured at regular time intervals (0, 2, 4, and 6 h) for all the four treated groups [25].

##### Assessment of Thermal Hyperalgesia

The thermal hyperalgesia was assessed in all the four treated-rat groups, following carrageenan-induced inflammation [26]. The temperature for the assessment of thermal hyperalgesia was kept at 50 ± 0.5 °C, and the cut-off time was selected at 35 s, to avoid any harm to the animals.

##### Tail Flick Test

The tail flick tests were performed to assess the nocifensive behavior (i.e., response to pain or discomfort) [26] of PTZ-loaded SLN2 (test sample), compared to the other treated-rat groups. For this purpose, the Wistar rats (20 rats, *n* = 5 per group) were positioned into separate cylindrical rat holders, leaving the tail hanging out freely. The animals got acclimatized to the holders for 30 min before testing. The 5 cm lower tail portion was marked. The marked part of tail was immersed in a cup of freshly filled water at 50 ± 0.5 °C. The reaction time was recorded using a stopwatch. After each determination, the tail was carefully dried. The reaction time was determined before and periodically at 0.5, 1, 2, 3, 4, and 6 h in all the four treated-rat groups. Rats that reacted in 5s were included in the study, and 15 s was used as a cut-off time to avoid tissue injury [27].

##### Tissue (Paw) Level Determination of Antioxidants and Oxidative Stress Markers

The carrageenan administration markedly elevates the oxidative stress and compromises the antioxidant defense system. The effect of PTZ-loaded SLN2 was assessed, compared to the other treated-rat groups, based on the production of antioxidants (i.e., GST, SOD, and catalase) and oxidative stress markers (i.e., MDA) in paw tissue, using the semi-quantitive ELISA (enzyme-linked immunoassay) method, as reported previously [28].

##### Tissue Level Determination of Pro-Inflammatory Cytokines

Pro-inflammatory cytokines are produced predominantly by activated macrophages and are critical players in the up-regulation of inflammatory reactions. There is abundant evidence that certain pro-inflammatory cytokines, such as IL-1β, IL-6, and TNF-α, are involved in the process of pathological pain. The effect of PTZ-loaded SLN2 was evaluated, compared to the other treated-rat groups, against the carrageenan-induced inflammatory cytokines, such as IL-1β, IL-6, and TNF-α. ELISA was performed to assess the concentration of the IL-1β, IL-6, and TNF-α in the paw tissue, following carrageenan-induced inflammation, at a dose of 5.0 mg/kg, as reported previously [29].

##### Histological Studies

The histopathology can provide insights of the tissue architecture and be used to assess tissue changes following inflammatory insult [30]. The hematoxylin and eosin (H&E) staining was performed to assess the effect of the SLNs on paw tissue. The tissue was removed, placed in formalin, embedded in paraffin, and, eventually, stained with HE. The histopathological changes were quantified according to a previously reported method [25].

### 2.10. Statistical Analysis

The data obtained from pharmacokinetic parameters and release rate were evaluated via the student *t*-test, using OriginPro 2018 (OriginLab Corporation, Northampton, MA, USA). All experiments were triplicated independently. All values were expressed as mean and standard deviation (mean ± SD). Statistically significant differences were assumed when *p* < 0.05 (* *p* < 0.05, ** *p* < 0.01, *** *p* < 0.001).

## 3. Results

### 3.1. Physicochemical Screening of SLNs and Selection of SLN2 as the Optimized Formulation

Two solid lipids cetyl alcohol and stearic acid were selected for SLNs formulation, by DoE (design of expert), on behalf of their dissolving capacity for PTZ, exhibiting a clear solution (molten state) in the presence of PTZ. DoE was used as the optimization software, for a reduction of experimental cost and time. The values were predicted by DoE for independent and dependent factors (Table 1 and Table S1). All the predicted values of dependent factors combined desirability DoE value of 0.738. Taguchi experimental design (L_8_), which refers to “off-line quality control”, reduced the number of experimental runs from 128 to 8 (Table 1).

The results obtained for the PTZ-loaded SLN2 (PS: 137.7 ± 1.01 nm; ZP: −16.60 ± 0.51 mV, PDI: 0.28 ± 0.005; EE: 86.00 ± 3.60%, and LC: 10.27 ± 0.50%), prepared by the emulsification–evaporation method, met most of the values predicted by DoE (Table 2). Since these values for PTZ-loaded SLN2 were overall the most suitable, SLN2 preparation was considered the optimized formulation and used for further experimental studies.

**Figure 1:**
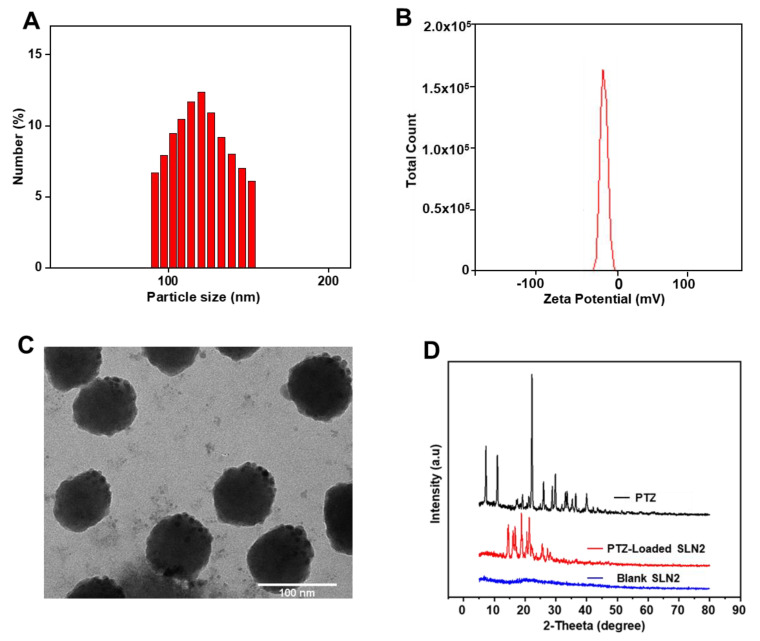
(**A**) Hydrodynamic diameter of optimized SLN_s_ formulation (SLN2) by DLS; (**B**) ZP of SLN2 formulation; (**C**) TEM micrograph of the SLN2 formulation. Scale bar is mentioned; (**D**) XRD spectrum of free PTZ (drug), blank SLNs (unloaded), and PTZ-loaded SLN2.

Thereby, PS was determined by measuring the random changes in the intensity of light scattered from the SLN formulation using DLS (dynamic light scattering); PS of the prepared PTZ-loaded SLNs ranged from 137.70 ± 1.01 nm to 261.40 ± 3.50 nm. PTZ-loaded SLN2 displayed a narrow particle size distribution (PSD) and the smallest PS (137.7 ± 1.01 nm), compared with the other prepared PTZ-loaded SLNs (Table 2 and Figure 1A). PS of blank SLN2 was expectedly smaller (101.6 ± 2.8 nm), due to the unloaded drug (data not shown), confirmed by the SLN dimension within the desirable size range (100–200 nm) required to enable Pickering functionality and a narrow PSD, which is usually encountered for blank SLNs prepared with such a method. Interestingly and concordantly, the PS of PTZ-loaded SLN2 (likewise PTZ-loaded SLN4, SLN7, and SLN8) did not show any significative difference, when compared with the optimized value (120.12 ± 10.49 nm) predicted with DoE (Table 2). It is also worth noting that the change of stearic acid to cetyl alcohol significantly (*p* < 0.05) decreased the average PS of the prepared SLNs.

ZP is a double layer surface electrostatic measure that offers a sign of colloidal system stability. ZP of the PTZ-loaded SLNs was negative and varied from −4.47 to −23.33 mV (Table 2). The ZP for PTZ-loaded SLN2, recorded as −16.60 ± 0.51 mV (Table 2 and Figure 1B), was selected as suitable for oral administration, one of the best of the prepared PTZ-loaded SLNs, along with PTZ-loaded SLN4 (−11.77 ± 0.50 mV) and PTZ-loaded SLN7 (−10.88 ± 0.37 mV), based on the lack of statistical difference with the optimized value (−13.52 ± 4.95), predicted with DoE (Table 2). The acetone/DCM ratio, lipid type, and time of homogenization (Table 1) were the most operative factors that displayed significant effects on ZP (*p* < 0.05). The overall results also revealed the influence of the solvents ratio, from 1:1 to 1:2, in the decrease of the absolute ZP value (*p* < 0.01).

PDI is defined as the standard deviation of the particle diameter distribution, divided by the mean particle diameter, and it is used to estimate the average uniformity of particle solution. Larger PDI values correspond to a larger PSD in the particle sample. While most of the PTZ-loaded SLNs exhibited PDI > 0.3, PTZ-loaded SLN2 formulation (along with PTZ-loaded SLN6 and SLN8) displayed a PDI < 0.3 (Table 2). The PDI values lying below 0.3 indicate uniform PSD. Thus, the suitability of PTZ-loaded SLN2 formulation and its method of preparation are once more confirmed.

In the pre-optimized SLN formulations, EE of PTZ ranged between 42.67 ± 1.52% and 86.00 ± 3.60%, with LC of PTZ (% of mass of the NP that is due to the encapsulated drug) ranging from 1.13 ± 0.12% to 10.27 ± 0.50% (Table 2). SLN2 appeared the most suitable lipid host matrix of appropriate dimensions that could enable the highest loading of a model hydrophobic/poorly hydrophilic active/drug (PTZ). Indeed, EE and LC values of SLN2 formulation were as high as 86.00 ± 3.60% and 10.27 ± 0.50%, respectively.

The morphological features of the SLNs, as well as precise PS, were analyzed carefully via TEM, a highly magnified imaging technique that uses a particle beam of electrons to visualize specimen at a high resolution. Thereby, TEM micrographs of SLN2 depicted round-shaped particles with a narrow size PSD, and the average PS of PTZ-loaded SLN2 was found to be 107 ± 5 nm (Figure 1A,C).

XRD relies on the dual wave/particle nature of X-rays to obtain information about the structure of crystalline materials. The 2-theta value represents the angle between transmitted beam and reflected beam. The structural behavior of PTZ, blank SLNs, and PTZ-loaded SLN2 was determined based on the nature of Bragg’s peaks that appeared in the respective XRD pattern (Figure 1D). The sharp peaks observed for PTZ and PTZ-loaded SLN2 (optimized formulation) indicate a crystalline behavior, while the broad humped peak blank SLNs indicates an amorphous behavior with short range ordering. These data demonstrate that PTZ in PTZ-loaded SLN2 maintained its crystalline behavior.

**Figure 2:**
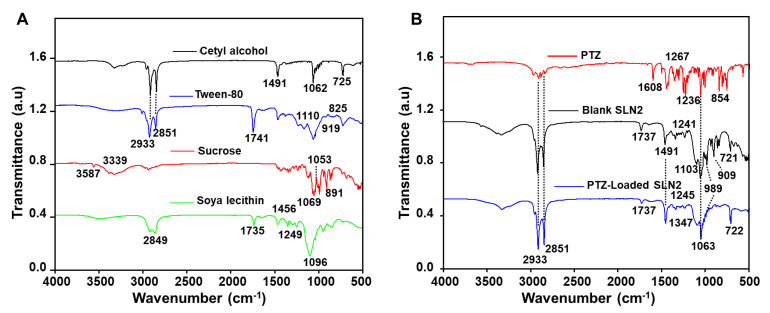
FTIR analysis of (**A**) cetyl alcohol, tween-80, sucrose, and soya lecithine; (**B**) PTZ (drug in its free form), blank-SLN2 (unloaded SNLs), and PTZ-loaded SLN2 (drug-loaded SLNs).

In a further step, FTIR assignments of the chemical reactions between the SLN2 excipients (Figure 2A) and the PTZ drug (Figure 2B) have been determined. Thereby, FTIR spectrum of pure cetyl alcohol identified the expected peaks at 725, 1062, and 1491 cm^−1^, which were attributed to –(CH2)*n*– (rocking), C–C vibrations, and C–H bend, respectively. The peaks confirm previous reported data on FTIR analysis of pure cetyl alcohol. FTIR spectrum of Tween-80 displayed characteristic peaks at 825 (C–O–O-stretch) and 919 cm^−1^ (sp^2^ C–H bend), as well as 1110 (C–O stretch), 1741.92 (C=O stretch), 2851 (C–H stretch), and 2923 cm^−1^ (C–H asymmetric/symmetric stretch). FTIR spectrum of sucrose revealed characteristic peaks attributed at 891 (C–O stretch), 1053 (C-O stretch), 1069 (C–O stretch), 3339 (OH stretch), and 3391 cm^−1^ (O-H stretch). The FTIR peaks of pure soya lecithin were shown at 1096 (C–H plane bend), 1249 (C–C stretch), 1456 (C–H bend), and 1735 cm^−1^ (strong C=O stretch). Different FTIR peaks were observed for the pure/free PTZ at the fingerprint region (400 to 1500 cm^−1^), which occurred specifically at 1608 (C=C-C stretch), 1267 (C–O stretch, 1236 cm^−1^ (C–O, acyl or phenyl stretch), 1067 (C-O, alkoxy stretch), and 854 cm^−1^ (sp^2^ C–H bend). The blank SLNs showed no interaction with the particles’ ingredients through characteristic peaks at 721 (C=C bending), 909.05 (C–H bend), 989 (C=C bend), 1064 (C–O stretch), 1103 (C–O stretch, secondary alcohol), 1241 (C–N stretch), and 1737 cm^−1^ (C=O stretch). PTZ-loaded SLN2 revealed peaks at 722, 2851, 2933 (strong C–H bend), 989 (C=C bend), 1063 (C–O stretch), 1103 (C-O stretch, secondary alcohol), 1245 (C–O stretch), 1347 (O-H bend stretch), 1491 (C–H bend), 1624 (C=C stretch), and 1737 cm^−1^ (C=O stretch). The purity of the peaks of PTZ indicated that there was no chemical reaction between the drug and the particles’ ingredients.

### 3.2. Stability of PTZ-Loaded SLN2

**Figure 3:**
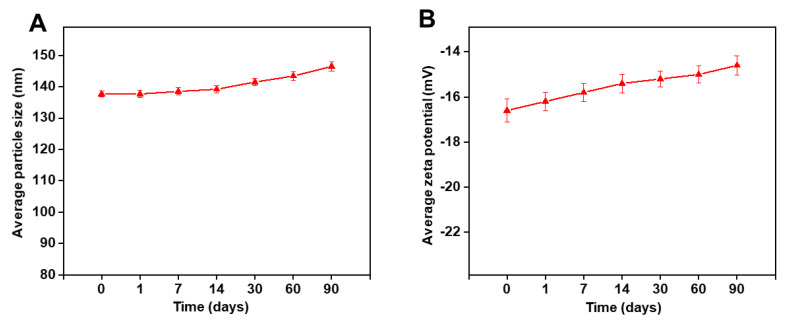
Stability of PTZ-loaded SLN2 over a period of 90 days. (**A**) Average PS; (**B**) average ZP, measured by DLS. Data are expressed as mean ± SD (n = 3). No significant differences were observed in PS and ZP over time (*p* > 0.05).

The stability studies were carried out on lyophilized SLN2, which appeared as a fine powder product (Figure S3). The average droplet PS (Figure 3A) and ZP (Figure 3B) of PTZ-loaded SLN2 were recorded for 3 months (0–90 days) at 25 °C. There were no significance changes in PS, ZP, and other (color) physicochemical properties over time (*p* > 0.05). Our data validate that the formulations have a long-term stability.

### 3.3. In Vitro PTZ Drug Release from PTZ-Loaded SLN2 (Simulated Conditions)

The greatest fit models of drug release kinetics were selected based on the constructions of regression coefficient (R^2^) nearby to 1 (Table S2). R^2^ values for first order was above the Zero order, which means that the drug release was concentration-dependent. In Higuchi model, the R^2^ values revealed that the drug release was diffusion controlled. The “n” values for Korsmeyer-Peppas model were found to be 0.49 and 0.65 at pH 1.2 and 6.8, respectively. This means that the drug release tails anomalous diffusion or non-Fickian mechanism associated with a characteristic diffusion process.

**Figure 4:**
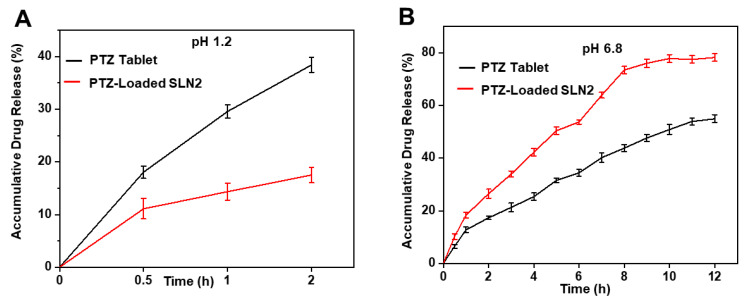
In vitro drug release profile of PTZ-loaded SLN2 and marketed PTZ tablets, using the dialysis-bag method in simulated conditions. (**A**) In SGF (0.1N HCl, pH 1.2) for 2 h; (**B**) in saliva-like condition (1× PBS, pH 6.8) for 12 h. Data are expressed as mean ± SD (n = 3). Mild statistical significance (*p* < 0.05) was observed during the first 30 min, while moderate statistical significance (*p* < 0.01) was observed from 2h.

PTZ-loaded SLN2 and free PTZ solutions were subjected to dissolution study in two different media, i.e., simulated gastric fluid (SGF, 0.1 N HCl, pH 1.2) (Figure 4A) and saliva-like condition (1× PBS, pH 6.8) (Figure 4B). The drug release profiles were sustained for 2 h in 0.1 N HCl, and for 12 h in PBS.

In SGF, PTZ-loaded SLN2 and PTZ in tablets showed an initial burst release during the first 30 min of 11.08 ± 1.9% and 18.04 ± 1.2% (*p* < 0.05), respectively; the same reached 17.5% and 38.41 ± 1.42% release within 2 h, respectively *p* < 0.01). The release of PTZ from SLN2 started reaching its maximum (plateau) at 2 h, indicating a limited release of PTZ from SLN2 in such a microenvironment, compared with PTZ from tablets.

In PBS (pH 6.8), PTZ-loaded in SLN2 and PTZ showed an initial burst release during the first hour of 18.5 ± 1.0% and 12.7 ± 1.0% (*p* < 0.05), respectively. Interestingly, PTZ loaded in SLN2 progressively reached 78.18 ± 1.4%, whereas only 54.89 ± 1.5% of PTZ was released from tablets within 12 h, *p* < 0.01).

Taken together, the data showed that, in a strong acidic microenvironment (e.g., stomach-like), PTZ from tablets is released much faster than PTZ loaded in SLN2, whereas in neutral microenvironment (e.g., blood-like), PTZ loaded in SLN2 is released in a much more controlled and sustained manner. These observations favor the use of PTZ-loaded SLN2 for *per os* (oral) administration.

### 3.4. Cytotoxicity and Cellular Permeability of PTZ-Loaded SLN2 in Caco-2 Cells

**Figure 5:**
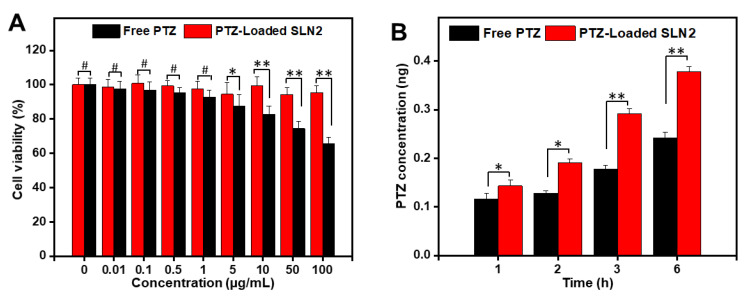
Evaluation of cytotoxicity and cellular permeation in Caco-2 cells. (**A**) Cellular cytotoxicity by MTT assay; PTZ-loaded SLN2 or free PTZ (used as control) were incubated with Caco-2 cells for 48 h, at the indicated concentrations (0–100 µg/mL); (**B**) transcellular transport/permeability of PTZ. Caco-2 cells were incubated with PTZ-loaded SLN2 (5 µg PTZ) or free PTZ (used as control) for different indicated times. Data are expressed as mean ± SD (n = 3). ^#^ *p* > 0.05 shows insignificant differences, whereas * *p* < 0.05, ** *p* < 0.01 are considered mild and moderate statistical significance, respectively.

The viability of Caco-2 cells treated for 48 h, with a wide range of concentrations of PTZ-loaded SLNs (0–100 µg/mL), was found to be concentration-independent and reached more than 90% in MTT assay (Figure 5A). This data indicates that PTZ-loaded SLN2 is a safe pharmaceutical formulation. Moreover, compared with the free drug, concentration from 5 μg/mL PTZ-loaded SLN2 is significantly less cytotoxic than free PTZ (Figure 5A), confirming the preferred choice of SLNs to boost the bioavailability of PTZ.

Besides, the Caco-2 cells monolayer model (which displays identical absorption properties than intestinal cells) was appropriate for the permeation studies after 17–21 days of incubation. When the cells monolayer showed stable value of over 500 Ω · cm^2^, then the transport mass of PTZ-loaded SLN2 and free PTZ was measured across transwell inserts after 1, 2, 3, and 6 h. The data show that the cell permeation of PTZ-loaded SLN2 formulation was, over time, significantly higher compared to that of free PTZ, again demonstrating the pertinence of using SLNs as a PTZ nanocarrier (Figure 5B).

### 3.5. Pharmacokinetic Studies in Wistar Rats

**Figure 6:**
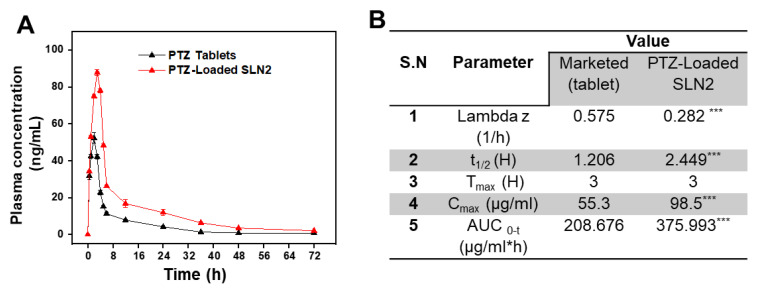
Pharmacokinetics profile of PTZ-loaded SLN2 and free PTZ marketed tablets. (**A**) Plasma concentration-time curve after a single oral dose (5 mg/kg) of PTZ-SLN2 in rats (n = 6), compared to free PTZ (5 mg/kg). Time points are indicated; (**B**) determination of pharmacokinetic parameters (n = 5). Data are expressed as mean ± SD (n = 3). *** *p* < 0.001 indicated high significant statistical difference.

The plasma concentration-time curve after a single oral dose (5 mg/kg) of PTZ-loaded SLN2 formulation in rats (n = 6) showed significant changes at specific the time points (0, 10, 30, 60, 120, 180, 240, 360, and 480 min) compared to that of the free PTZ marketed tablet (Figure 6A). Based on the pharmacokinetic parameters after oral administration of PTZ-loaded SLN2 versus free PTZ, the relative bioavailability of PTZ-loaded SLN2 was found to be approximately two-fold higher compared to that of free PTZ (Figure 6B). Additionally, the average Area Under the (Moment) curve (AUC)_0–t_ of free PTZ and PTZ-loaded SLN2 was 208.68 ± 11.47 µg.h/mL and 375.99 ± 27.73 µg.h/mL, respectively, which showed significant improvement in the AUC of PTZ when loaded to SLN2 (*p* < 0.001). The mean residence time (MRT) and t_1/2_ observed for the PTZ-loaded SLN2 showed significant improvement (*p* < 0.001), when compared to the free PTZ marketed formulation.

### 3.6. Carrageenan-Induced Paw Edema, Thermal Hyperalgesia, and Tail Flick Response in Wistar Rats

**Figure 7:**
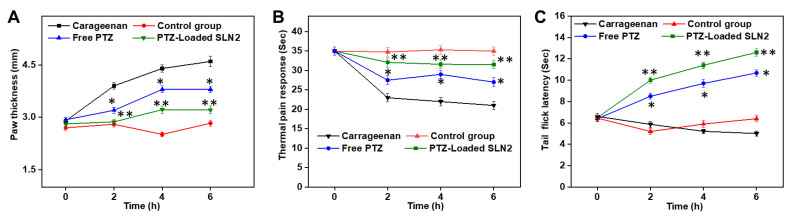
Effects of PTZ-SLN2 on the paw edema of Wistar rats (N = 20 rats, n = 5/group) following carrageenan-induced inflammatory pain. The experiments were conducted for 6 h. (**A**) Impact on paw thickness (mm); (**B**) Impact on thermal analgesia (sec); (**C**) Impact on tail flick latency (sec). Control group 1 (1× PBS, orally administrated), carrageenan group 2 (200 μL/paw of the 1% *w*/*v* solution, single injection), free PTZ group 3 (carrageenan 200 μL/paw of the 1% *w*/*v* solution + free PTZ orally administrated at 5 mg/kg), and PTZ-loaded SLN2 group 4 (carrageenan 200 μL/paw of the 1% *w*/*v* solution + PTZ-loaded SLN2 orally administrated at 5 mg/kg). The carrageenan was administered 30 min into the Groups 3 and 4 before the treatment (with free PTZ and PTZ-loaded SLN2, respectively). The carrageenan group showed a drastic increase in the paw thickness and hyperalgesia when compared to the (vehicle) control group, which were significantly reduced in the free PTZ and, in a larger extent, PTZ-SLN2 groups. Data are expressed as mean ± SD (n = 3). * *p* < 0.05 and ** *p* < 0.01 represent mild and moderate statistical significance, respectively, when compared to the carrageenan group.

The single i.pl injection of carrageenan in rats has markedly elevated the paw inflammation in rats compared to that of vehicle control group (i.e., PBS) (Figure 7A). This significant increase based on the paw thickness was noticed over the course of the period study (up to 6 h). However, the free PTZ (*p* < 0.05) group and, most noticeably, PTZ-loaded SLN2 group (*p* < 0.01) showed a significant reduction in paw edema over time, compared to the carrageenan group (Figure 7A). Further, this effect caused by PTZ-loaded SLN2 was insignificantly different (*p* > 0.05) at 6 h, when compared to that of the (vehicle) control group (Figure 7A).

The thermal analgesia (pain) response was drastically enhanced following a single i.pl administration of carrageenan in rats compared to that of vehicle control group (Figure 7B). This significant increase (*p* < 0.001) was noticed over the course of the period study (up to 6 h). Meantime, both the free PTZ (*p* < 0.05) and PTZ-loaded SLN2 markedly (*p* < 0.01) decreased the thermal pain response, compared to that of the carrageenan group, and this response was quite comparable, when compared to that of the control group (*p* > 0.05) (Figure 7B).

The tail flick response (thermal hyperalgesia) was unaltered following a single i.pl administration of carrageenan in rats compared to that of (vehicle) control group (Figure 7C). This insignificant change (*p* > 0.05) in tail flick latency was noticed over the course of the period study (up to 6 h). However, in the PTZ and PTZ-loaded SLN2 groups, the antinociceptive response was markedly increased over time (*p* < 0.05 and *p* < 0.01, respectively), compared to that of the carrageenan group (Figure 7C).

Overall, it is worth noting that PTZ-loaded SLN2 could revert substantially the carrageenan-induced pain and inflammation.

### 3.7. Carrageenan-Induced Oxidative stress in Wistar Rats

In a step forward, the antioxidant systems and oxidative stress levels were analyzed in the animals, and the samples were collected for analyses 6 h after treatment for ELISA measurements.

**Figure 8:**
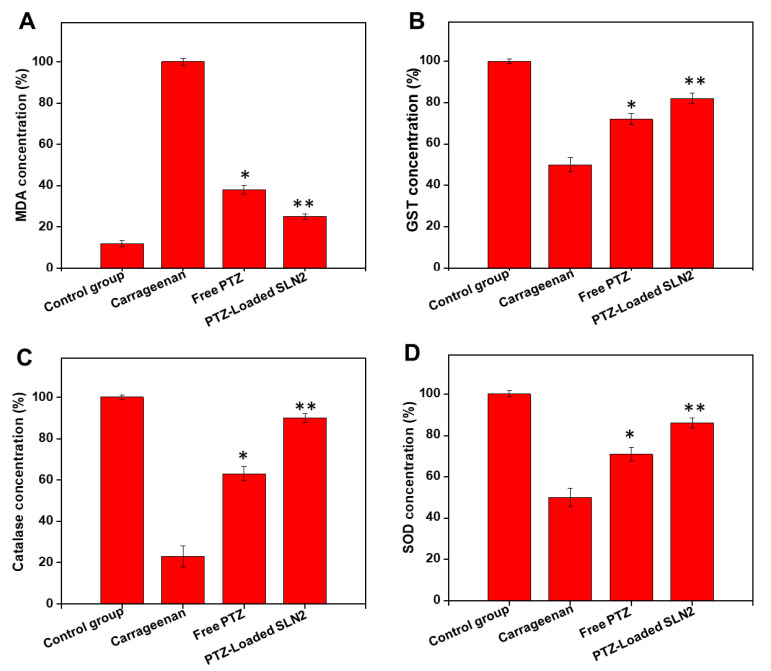
Effects of PTZ-SLN2 on the antioxidants and oxidative stress markers in the paw tissue of Wistar rats (N = 20 rats, n = 5/group), following carrageenan-induced inflammatory pain. The samples were analyzed by ELISA, at the time point of 6 h after treatment. (**A**) Impact on MDA concentration (%); (**B**) impact on GST concentration (%); (**C**) impact on catalase concentration (%); (**D**) impact on SOD concentration (%). Control group 1 (1×PBS, orally administrated), carrageenan group 2 (200 μL/paw of the 1% *w*/*v* solution, single injection), free PTZ group 3 (carrageenan 200 μL/paw of the 1% *w*/*v* solution + free PTZ, orally administrated at 5 mg/kg), and PTZ-loaded SLN2 group 4 (carrageenan 200 μL/paw of the 1% *w*/*v* solution + PTZ-loaded SLN2, orally administrated at 5 mg/kg). The carrageenan was administered into the Groups 3 and 4, 30 min before the treatment (with free PTZ and PTZ-loaded SLN2, respectively). The carrageenan group showed marked increase in the stress oxidative-induced MDA concentration and a significant decrease in the antioxidant systems (i.e., GST, catalase, and SOD), compared to the (vehicle) control group (*p* < 0.001). These effects were all significantly reduced in the free PTZ and, to a better extent, PTZ-SLN2 groups. Data are expressed as mean ± SD (*n* = 3). * *p* < 0.05 and ** *p* < 0.01 represent mild and moderate statistical significance, respectively, when compared to the carrageenan group.

The single i.pl injection of carrageenan drastically elevated the oxidative stress marker MDA in the paw tissue (plantar tissue) of the rats (Figure 8A). However, free PTZ and, to a higher extent, PTZ-loaded SLN2 significantly decreased the carrageenan-induced MDA levels (*p* < 0.05 and *p* < 0.01, respectively).

Conversely, the carrageenan administration significantly decreased the enzymatic antioxidants glutathione S-transferase (GST) (Figure 8B), catalase (Figure 8C), and superoxide dismutase (SOD) (Figure 8D) in the paw tissue of the rats. However, PTZ and, to a better extent, PTZ-loaded SLN2 increased the antioxidants enzymes significantly (*p* < 0.05 and *p* < 0.01, respectively), compared to the carrageenan-treated group.

Overall, PTZ-loaded SLN2 could substantially revert the carrageenan-induced oxidative stress, although not completely (*p* < 0.05), when comparisons are made to the (vehicle) control (Figure 8A–D).

### 3.8. Carrageenan-Induced Pro-Inflammatory Cytokines in Wistar Rats

To possibly corroborate the previous data obtained on carrageenan-induced inflammatory pain, the levels of pro-inflammatory cytokines, like IL-6, IL-1β, and TNF-α were analyzed in the animals. The samples were collected for analyses 6 h after treatment for ELISA measurements.

**Figure 9:**
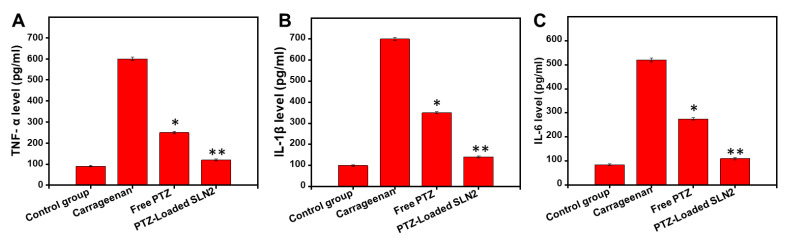
Effects of PTZ-SLN2 on the inflammatory cytokines in paw tissue of Wistar rats (N = 20 rats, *n* = 5/group) following carrageenan-induced inflammatory pain. The samples were analyzed by ELISA at the time point of 6 h after treatment. (**A**) Impact on TNF-α concentration (pg/mL); (**B**) Impact on IL-1β concentration (pg/mL); (**C**) Impact on IL-6 concentration (pg/mL). Control group 1 (1× PBS, orally administrated), carrageenan group 2 (200 μL/paw of the 1% *w*/*v* solution, single injection), free PTZ group 3 (carrageenan 200 μL/paw of the 1% *w*/*v* solution + free PTZ orally administrated at 5 mg/kg), and PTZ-loaded SLN2 group 4 (carrageenan 200 μL/paw of the 1% *w*/*v* solution + PTZ-loaded SLN2, orally administrated at 5 mg/kg). The carrageenan was administered 30 min into the Groups 3 and 4 before the treatment (with free PTZ and PTZ-loaded SLN2, respectively). The carrageenan group strongly increased the tested pro-inflammatory cytokines, compared to that of the (vehicle) control group. These effects were all significantly reduced in the free PTZ group and, to a better extent, PTZ-loaded SLN2 group. Data are expressed as mean ± SD (*n* = 3). * *p* < 0.05 and ** *p* < 0.01 represent mild and moderate statistical significance, respectively, when compared to the carrageenan group.

Compared to that of the (vehicle) control group, the concentration levels of IL-6, IL-1β, and TNF-α, were significantly increased (about 6-fold in average, *p* < 0.001) following a single i.pl injection of carrageenan in the rats (Figure 9A–C). However, the free PTZ group showed marked reduction in the inflammatory cytokines compared to the carrageenan group (*p* < 0.05). Interestingly, the PTZ-loaded SLN2 group further reduced the production of all tested inflammatory cytokines compared to the carrageenan group (*p* < 0.01) (Figure 9A–C).

### 3.9. PTZ-Loaded SLN2 Reverts the Carrageenan-Induced Histological Changes in Wistar Rats

**Figure 10:**
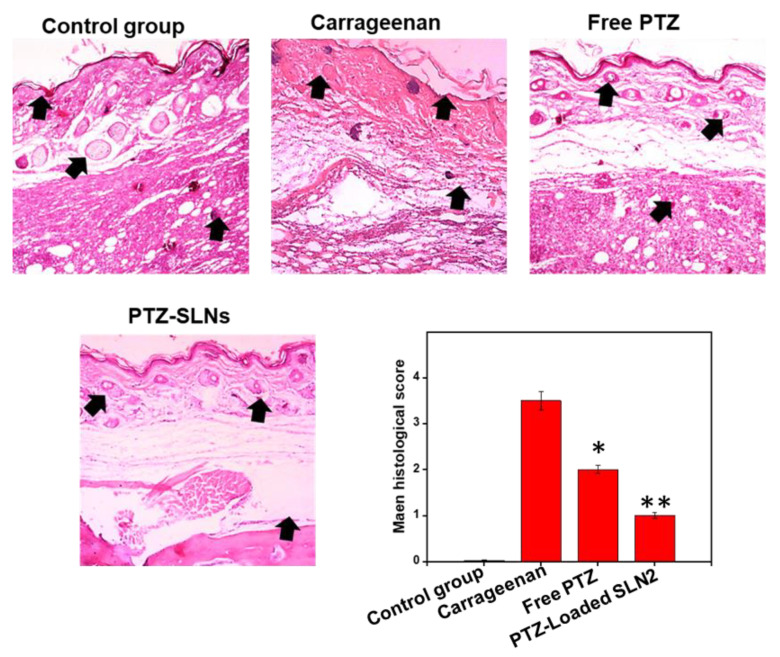
Effects of PTZ-SLN2 on the paw tissue architecture of Wistar rats (N = 20 rats, *n* = 5/group), following carrageenan-induced inflammatory pain. The samples were analyzed after H&E staining at the time point of 6 h after treatment. Control group 1 (1×PBS, orally administrated), carrageenan group 2 (200 μL/paw of the 1% *w*/*v* solution, single injection), free PTZ group 3 (carrageenan 200 μL/paw of the 1% *w*/*v* solution + free PTZ, orally administrated at 5 mg/kg), and PTZ-loaded SLN2 group 4 (carrageenan 200 μL/paw of the 1% *w*/*v* solution + PTZ-loaded SLN2, orally administrated at 5 mg/kg). The carrageenan was administered 30 min into the Groups 3 and 4 before the treatment (with free PTZ and PTZ-loaded SLN2, respectively). The carrageenan group strongly altered the paw tissue architecture, compared to the control group. These effects were all significantly reduced in the free PTZ and, to a better extent, PTZ-SLN2 groups, when compared to the carrageenan group. Data are expressed as mean ± SD (*n* = 3). * *p* < 0.05 and ** *p* < 0.01 represent mild and moderate statistical significance, respectively, when compared to the carrageenan group.

Potential paw tissue changes were assessed by H&E staining 6 h after treatment, following a single i.pl injection of carrageenan (Figure 10 and Figure S4). The carrageenan group displayed markedly altered changes of the paw tissue architecture, compared to the vehicle control group (*p* < 0.001). However, the free PTZ (*p* < 0.05) and, to a better extent, PTZ-loaded SLN2 group (*p* < 0.01) showed remarkable improvement in the i.pl histology, compared to the carrageenan group (Figure 10).

## 4. Discussion

SLNs are considered an excellent choice for oral administration of poorly water-soluble and unstable drugs [27]. Indeed, SLNs enhance bioavailability by shielding such drugs from denaturation in the gastrointestinal lumen and extending the exposure of the mucous membrane to raise drug concentration [9]. Thereby, SLNs can overcome drug complications, such as low solubility, low mucosal permeability, first-pass hepatic metabolism, and gut metabolism [3,27].

In the oral delivery of SLNs, NPs are taken up at the surface of specialized epithelial cells, namely the M cells located in the follicle-associated epithelium of the gastrointestinal tract and transported to lymphocytes in the form of vesicles. M cells have a high capacity for transcytosis of a wide range of microorganisms and macromolecules and, thus, are believed to act as an antigen sampling system [3,9,31].

In this study, in vitro physicochemical properties (i.e., PS, PDI, ZP, surface morphology, structural interactions, and nature), in vitro pharmacological analyses (i.e., EE, LC, drug release, and pharmacokinetic parameters), and in vivo (in Wistar rats) biological evaluations (i.e., carrageenan-induced paw edema, thermal hyperalgesia, tail flick latency, oxidative stress, antioxidant activity, and pro-inflammatory cytokines) of formulated PTZ-loaded SLNs versus free PTZ marketed drug (5 mg/kg) were assessed.

From 8 different SLNs, PTZ-SLN2 was selected as the optimal formulation, based on PS and LC. PS, or the diameter of SLNs, is defined as a critical parameter for oral bioavailability of lipophilic drugs and significantly influences the absorption and, subsequently, the availability of drug at site of action [32]. The results of the current study demonstrated that cetyl alcohol SLNs gives a smaller size, compared to stearic acid, and this may be due to the stearic acid long C18 carbon chain, while cetyl alcohol is represented by a 16C chain. Increase in surfactant concentration was also effective in producing a smaller size of SLNs (up to 3%), indicating the +ve effect of surfactant on PS [32]. Generally, smaller PS values are observed when a higher surfactant/lipid ratio was selected, while a decrease in the surfactant amount results in PS increase, which is most likely due to the increased drug LC [16]. Interestingly, soya lecithin, used as co-surfactant as internal emulsifier in SLNs and hydrogels [33], favored the PS reduction and stability, with a good PDI, confirming the previously reported data [18].

The average PS by TEM of PTZ-loaded SLN2 was about 107 ± 5 nm, with round-shaped geometry and narrow PSD. The average hydrodynamic PS and PDI for the optimized formulation SLNs, measured by DLS, were recorded as 137.7 ± 5.2 nm and 0.28, respectively; the average of PS for SLN2 lied in the acceptable range, between 100 nm and 200 nm, and a PDI value lying below 0.3 is generally considered good, which shows uniform PSD [18]. The hydrodynamic PS is found to be slightly bigger than PS measured by TEM, which may be due the hydration layer around the SLNs in aqueous medium [3].

ZP is a surface electrostatic double layer value, a key factor to understand the application of the dispersion and aggregation processes; it is certainly an important criterion for studying the storage stability in NPs. ZP of all SLN formulations were negative and ranged between −4.47 and −23.33 mV. The predicted value of ZP was close to the optimized SLNs. The results revealed that changing the acetone/DCM from a 1:1 to 1:2 ratio significantly decreased the absolute value of the ZP. ZP for SLN2 was recorded as −16.60 mV by DLS, which is considered good for oral SLNs. Although a high concentration of surfactant reduced the PS of SLNs, it may result in a decrease of EE and cause toxic effects [30].

The contour plot of LC showed that, at a low speed of stirring (less than 925 rpm) and surfactant (3%), the drug LC increases, while the low level of acetone:DCM ratios, with a high level of stirring rates, caused the highest LC efficiencies, which is consistent with previous observations [16]. However, above the optimum level (i.e., 3%), the highest % used in this study, the surfactant is known to cause a decrease in LC [9].

XRD data revealed that PTZ-loaded SLN2 were crystalline in nature, and that PTZ maintained this crystallinity after its loading in the SLNs. The crystalline form of a drug, in such a formulation, confirms the stability of the formulation [11].

Characteristic FTIR bands may fall over a range of wavenumbers (cm^−1^), and specific substituents (functional groups) may cause variations in absorption frequencies (functional peaks). Functional peaks of formulation ingredients were consistent with the reported data [11,34,35,36]. Peaks in blank (unloaded SLN2), free PTZ, and loaded SLN2 confirmed that PTZ was successfully entrapped in the SLNs and did not interact with the excipients. It is largely assumed by the scientific community that drug interactions with excipients generally causes incompatibilities and a decrease in therapeutic efficacy [37].

In vitro studies revealed a minimal cytotoxicity in Caco-2 cells (immortalized cell line of human colorectal adenocarcinoma cells), compared to the marketed formulation (free PTZ). Drug toxicity is an important parameter to avoid safety concerns, and mainly depends on the drug bioavailability [2,20]. Interestingly, the results revealed that PTZ-loaded SLN2, passing via the strong acidic stomach-like environment (GSG, pH 1.2), tends to quickly release most of the amount of the PTZ drug, whereas in neutral-like conditions (PBS, pH 6.8), the PTZ release from SLN2 occurred in a controlled manner after an initial burst release. The first burst release in acidic media may be due to drug entrapment in the outer area of the SLNs. Additionally, it is worth mentioning that the marketed free PTZ is more sensitive to both microenvironments, thereby favoring the oral administration of PTZ encapsulated into SNLs. Further, the enhanced permeation capability of the PTZ-loaded SLNs, carried out using Caco-2 cells and considered the most appropriate model of the intestinal epithelial barrier [38,39], can be explained by their sustained release behavior and lipophilic nature, compared to the marketed free PTZ [32]. Thus, the improved permeation of SLNs again prove their use as an effective carrier for the oral delivery of PTZ for improved bioavailability in the systemic circulation. Indeed, such lipid-based delivery systems can promote absorption and enhance the solubility of hydrophobic drugs [8].

In vivo pharmacokinetics data confirmed the enhanced oral bioavailability in male Wistar rats of PTZ-loaded SLNs (up to two-fold, compared to that of free PTZ marketed tablets), with a steady-state concentration of PTZ (5 mg/kg). The increase of PTZ drug bioavailability from SLNs can be explained by the first-pass effect. Indeed, due to the lymphatic uptake, the drug bypassed from the first-pass (presystemic) metabolism—a phenomenon whereby the concentration of a drug is greatly reduced before it reaches the systemic circulation—and, subsequently, results in increased (intestinal) absorption and (blood/tissues) bioavailability [9].

An established carrageenan-induced inflammatory pain model [25] was used in this study for the assessment of anti-inflammatory and analgesic activities of PTZ-loaded SLN2. It is now admitted that carrageenan administration and/or consumption triggers inflammation by inducing multiple signaling mechanisms, which lead to the production of pro-inflammatory cytokines (e.g., IL-1β, IL-6, and TNF-α) and cause(s) oxidative stress by compromising the antioxidant systems (e.g., GST, GSH, catalase, and SOD) of the body [25]. These noxious stimuli reduce the threshold for analgesia by stimulating the nociceptors and production of painful sensation [25]. Importantly, PTZ-loaded SLN2, evaluated against the carrageenan-induced inflammatory pain model, clearly showed beneficial effects against inflammation, paw edema, oxidative stress, and hyperalgesia activity. Histopathological findings from paw tissue, as well as from liver tissue (Figure S5), confirmed the efficacy and safety of using PTZ-loaded SLN2, compared to free PTZ.

## 5. Conclusions

The occurrence of low systemic availability, due to significant first-pass metabolism, leading to poor absorption of orally administered drugs, such as the narcotic analgesic/pain medicine PTZ, has been well-recognized. SLNs are considered a useful DDS for hepatically metabolized drugs, both to avoid the first-pass metabolism and allow the lymphatic uptake of orally administered drugs. This study strongly demonstrates that PTZ-loaded SLNs is an effective DDS, not only to enhance the bioavailability of the conventional marketed PTZ but also to safely reduce the inflammation and associated pain.

## Figures and Tables

**Table 1:** Predicted independent variables (N = 7) by DoE in the preparation of SLNs.

SLNs Code	Lipid Type	Surfactant Type	Surfactant (%)	Acetone: DCM	Lecithin: Lipid	HG Speed (rpm)	HG Time (min)
SLN1	Stearic acid	Tween 20	3	1:2	2:7	15,000	15
SLN2	Cetyl alcohol	Tween 80	3	1:2	1:8	16,000	15
SLN3	Cetyl alcohol	Tween 80	2	1:2	2:7	16,000	10
SLN4	Stearic acid	Tween 20	2	1:1	2:7	16,000	15
SLN5	Stearic acid	Tween 20	2	1:2	1:8	15,000	10
SLN6	Cetyl alcohol	Tween 80	2	1:1	1:8	15,000	15
SLN7	Cetyl alcohol	Tween 20	3	1:1	1:8	16,000	10
SLN8	Stearic acid	Tween 80	3	1:1	2:7	15,000	10

SLNs: Solid lipid nanoparticles (blank/unloaded); DCM: dichloromethane; HG: homogenization.

**Table 2:** SLNs-dependent variables respective to DoE-predicted independent variables.

Code	PS (nm)	ZP (mV)	PDI	EE%	LC%
**Experimental** SLN1	189.40 ± 2.05	−4.47 ± 0.35	0.44 ± 0.04	68.67 ± 0.57	1.70 ± 0.30
SLN2	137.70 ± 1.01	−16.60 ± 0.51	0.28 ± 0.005	86.00 ± 3.60	10.27 ± 0.50
SLN3	249.70 ± 1.65	−23.33 ± 0.90	0.38 ± 0.015	42.67 ± 1.52	1.13 ± 0.12
SLN4	172.00± 8.18	−11.77 ± 0.50	0.57 ± 0.064	75.67 ± 2.08	1.30 ± 0.26
SLN5	237.00 ± 4.05	−5.33 ± 0.30	0.53 ± 0.025	47.47 ± 1.50	2.37 ± 0.32
SLN6	261.40 ± 3.50	−19.32 ± 0.91	0.23 ± 0.015	78.33 ± 1.52	1.77 ± 0.20
SLN7	154.70 ± 3.80	−10.88 ± 0.37	0.74 ± 0.040	70.67 ± 2.09	4.03 ± 0.20
SLN8	146.80 ± 3.29	−5.39 ± 0.63	0.17 ± 0.035	43.67 ± 3.21	2.73 ± 0.70
Predicted SLNs	120.12 ± 10.49	−13.52 ± 4.95	0.45 ± 0.07	61.87 ± 5.75	25.00 ± 3.98

SLN: Solid lipid nanoparticle (PTZ-loaded); PS: particle size; ZP: zeta potential; PDI: polydispersity index; EE: encapsulation efficiency; LC: loading capacity; same letters mean no statistical difference in SLNs for a given variable (*p* < 0.05).

## Data Availability

All the data published (or not) in this manuscript are available upon request for academic or industrial use/collaboration.

## Supplementary Materials

The following are available online at https://www.mdpi.com/article/10.3390/pharmaceutics14020409/s1, Figure S1: Fine powder of lyophilized SLN2; Figure S2: Chromatographic profile of free PTZ, Figure S3: Effects of the PTZ-loaded SLN2 on the paw tissue architecture following carrageenan-induced inflammatory pain in Wistar rats, Figure S4: Histological effects of PTZ-loaded SLN2 on the liver of Wistar rats, Table S1: Independent variables predicted by DoE, Table S2: PTZ drug release models at pH 1.2 and 6.8.

## References

[1] Rajpoot K. (2019). Solid Lipid Nanoparticles: A Promising Nanomaterial in Drug Delivery. Curr. Pharm. Des..

[2] Menaa F., Menaa B. (2012). Development of mitotane lipid nanocarriers and enantiomers: Two-in-one solution to efficiently treat adreno-cortical carcinoma. Curr. Med. Chem..

[3] Hou D.Z., Xie C.S., Huang K.J., Zhu C.H. (2003). The production and characteristics of solid lipid nanoparticles (SLNs). Biomaterials.

[4] Duan Y., Dhar A., Patel C., Khimani M., Neogi S., Sharma P., Kumar N.S., Vekariya R.L. (2020). A brief review on solid lipid nanoparticles: Part and parcel of contemporary drug delivery systems. RSC Adv..

[5] Hilda A., Nashiru B. (2021). Lyophilized Drug-Loaded Solid Lipid Nanoparticles Formulated with Beeswax and Theobroma Oil. Molecules.

[6] Menaa F. (2013). When pharma meets nano or the emerging era of nano-pharmaceuticals. Pharm. Anal. Acta.

[7] Mehnert W., Mäder K. (2012). Solid lipid nanoparticles: Production, characterization and applications. Adv. Drug Deliv. Rev..

[8] Bhalekar M.R., Upadhaya P., Madgulkar A.R., Kshirsagar S.J., Dube A., Bartakke U.S. (2016). *In-vivo* bioavailability and lymphatic uptake evaluation of lipid nanoparticulates of darunavir. Drug Deliv..

[9] Mishra A., Vuddanda P.R., Singh S. (2014). Intestinal lymphatic delivery of praziquantel by solid lipid nanoparticles: Formulation design, in vitro and in vivo studies. J. Nanotechnol..

[10] Mahapatra S.J., Jain S., Bopanna S., Gupta S., Singh P., Trikha A., Sreenivas V., Shalimar, Garg P.K. (2019). Pentazocine, a kappa-opioid agonist, is better than diclofenac for analgesia in acute pancreatitis: A randomized controlled trial. Am. J. Gastroenterol..

[11] Madni A., Rahim M.A., Mahmood M.A., Jabar A., Rehman M., Shah H., Khan A., Tahir N., Shah A. (2018). Enhancement of dissolution and skin permeability of pentazocine by proniosomes and niosomal gel. AAPS PharmSciTech..

[12] Eleje G.U., Egeonu R.O., Obianika C., Mbachu I., Okohue J., Osuagwu I. (2015). Diclofenac and pentazocine versus pentazocine alone for post-operative analgesia in cesarean section. Int. J. Med. Health Dev..

[13] Verma P.P., Chandak A. (2009). Development of matrix controlled transdermal delivery systems of pentazocine: In vitro/in vivo performance. Acta Pharm..

[14] Makoni P.A., Ranchhod J., Kasongo K.W., Khamanga S.M., Walker R.B. (2020). The use of quantitative analysis and Hansen solubility parameter predictions for the selection of excipients for lipid nanocarriers to be loaded with water soluble and insoluble compounds. Saudi Pharm. J..

[15] Varshosaz J., Tabbakhian M., Mohammadi M.Y. (2010). Formulation and optimization of solid lipid nanoparticles of buspirone HCl for enhancement of its oral bioavailability. J. Liposome Res..

[16] Furuishi T., Io T., Fukami T., Suzuki T., Tomono K. (2008). Formulation and in vitro evaluation of pentazocine transdermal delivery system. Biol. Pharm. Bull..

[17] Carlotti M.E., Sapino S., Trotta M., Battaglia L., Vione D., Pelizzetti E. (2005). Photostability and stability over time of retinyl palmitate in an O/W emulsion and in SLN introduced in the emulsion. J. Dispers. Sci. Technol..

[18] He H., Wang P., Cai C., Yang R., Tang X. (2015). VB12–coated Gel-Core-SLN containing insulin: Another way to improve oral absorption. Int. J. Pharm..

[19] Carvajal-Vidal P., Fábrega M.J., Espina M., Calpena A.C., García M.L. (2019). Development of Halobetasol-loaded nanostructured lipid carrier for dermal administration: Optimization, physicochemical and biopharmaceutical behavior, and therapeutic efficacy. Nanomed. Nanotechnol. Biol. Med..

[20] Iqbal H., Khan B.A., Khan Z.U., Razzaq A., Khan N.U., Menaa B., Menaa F. (2020). Fabrication, physical characterizations and in vitro antibacterial activity of cefadroxil-loaded chitosan/poly(vinyl alcohol) nanofibers against *Staphylococcus aureus* clinical isolates. Int. J. Biol. Macromol..

[21] Verhoeckx K., Cotter P., López-Expósito I., Kleiveland C., Lea T., Mackie A., Requena T., Swiatecka D., Wichers H. (2015). The Impact of Food Bioactives on Health: In Vitro and Ex Vivo Models.

[22] Dayana K., Manasa M.R. (2019). Comparative study of analgesic activity of *Lagenaria siceraria* root extract with pentazocine in albino mice. Nat. J. Physiol. Pharm. Pharmacol..

[23] Jones K. (2015). Oral Dosing (Gavage) in Adult Mice and Rats SOP. UBC Anim. Care Comm..

[24] Eleesha S., Mahira Z., Qurat U.A., Ashraf U.K., Irshad H., Salman K., Elise L., Hussain A. (2021). Topical delivery of curcumin-loaded transfersomes gel ameliorated rheumatoid arthritis by inhibiting NF-κβ pathway. Nanomedicine.

[25] Rasheed H., Afridi R., Khan A.U., Ullah M.Z., Khalid S., Atiq A., Kashif H., Ahmed M.N., Kim Y.S., Khan S. (2018). Anti-inflammatory, anti-rheumatic and analgesic activities of 2-(5-mercapto-1,3,4-oxadiazol-2-yl)-*N*-propylbenzenesulphonamide (MOPBS) in rodents. Inflammopharmacology.

[26] Khalid S., Ullah M.Z., Khan A.U., Afridi R., Rasheed H., Khan A., Ali H., Kim Y.S., Khan S. (2018). Antihyperalgesic Properties of Honokiol in Inflammatory Pain Models by Targeting of NF-κB and Nrf_2_ Signaling. Front. Pharmacol..

[27] Khan A., Ullah M.Z., Afridi R., Rasheed H., Khalid S., Ullah H., Ali H., AlSharari S.D., Kim Y.S., Khan S. (2019). Antinociceptive properties of 25-methoxy hispidol A, a triterpinoid isolated from *Poncirus trifoliata* (Rutaceae) through inhibition of NF-κB signalling in mice. Phytother. Res..

[28] Chatterjee S., Dziubla T., Butterfield D.A. (2016). Chapter Two—Oxidative Stress, Inflammation, and Disease. Oxidative Stress and Biomaterials.

[29] Kapoor M., Martel-Pelletier J., Lajeunesse D., Pelletier J.-P., Fahmi H. (2011). Role of proinflammatory cytokines in the pathophysiology of osteoarthritis. Nat. Rev. Rheumatol..

[30] Pearse G. (2006). Histopathology of the thymus. Toxicol. Pathol..

[31] Kucharzik T., Lügering N., Rautenberg K., Schmidt M.A., Stoll R., Domschke W. (2000). Role of M Cells in Intestinal Barrier Function. Ann. N. Y. Acad. Sci..

[32] Eman S., Mahmoud M.A., Yuanhu P., Dongmei C., Shuyu X. (2020). Solid lipid nanoparticles for enhanced oral absorption: A review. Colloids Surf. B Biointerfaces.

[33] Singh V.K., Pandey P.M., Agarwal T., Kumar D., Banerjee I., Anis A., Pal K. (2016). Development of soy lecithin based novel self-assembled emulsion hydrogels. J. Mech. Behav. Biomed. Mater..

[34] Wembabazi E., Mugisha P.J., Ratibu A., Wendiro D., Kyambadde J., Vuzi P.C. (2015). Spectroscopic analysis of heterogeneous biocatalysts for biodiesel production from expired sunflower cooking oil. J. Spectrosc..

[35] Nazief A.M., Hassaan P.S., Khalifa H.M., Sokar M.S., El-Kamel A.H. (2020). Lipid-Based Gliclazide Nanoparticles for Treatment of Diabetes: Formulation, Pharmacokinetics, Pharmacodynamics and Subacute Toxicity Study. Int. J. Nanomed..

[36] Rizvi S.Z.H., Shah F.A., Khan N., Muhammad I., Ali K.H., Ansari M.M., Ud Din F., Qureshi O.S., Kim K.-W., Choe Y.-H. (2019). Simvastatin-loaded solid lipid nanoparticles for enhanced anti-hyperlipidemic activity in hyperlipidemia animal model. Int. J. Pharm..

[37] Gorain B., Choudhury H., Pandey M., Madheswaran T., Kesharwani P., Tekade R.K., Tekade R.K. (2018). Chapter 11—Drug-Excipient Interaction and Incompatibilities. Advances in Pharmaceutical Product Development and Research, Dosage Form Design Parameters.

[38] Degim Z., Unal N., Essiz D., Abbasoglu U. (2004). Caco-2 Cell Culture as a Model for Famotidine Absorption. Drug Deliv..

[39] Lea T., Verhoeckx K., Cotter P., López-Expósito I., Kleiveland C., Lea T., Mackie A., Requena T., Swiatecka D., Wichers H. (2015). Caco-2 Cell Line. The Impact of Food Bioactives on Health: In Vitro and Ex Vivo Models.

