# Supplementary material for: Physicochemical Characterizations and Pharmacokinetic Evaluation of Pentazocine Solid Lipid Nanoparticles against Inflammatory Pain Model
Source: Pharmaceutics. 2022 Feb 14;14(2):409. doi: 10.3390/pharmaceutics14020409 (PMC8876599; doi:10.3390/pharmaceutics14020409)
Supplement: Supplementary file 1 [file pharmaceutics-14-00409-s001.zip › pharmaceutics-1522124-SI.pdf]

pharmaceutics

MDPI

Supplementary Materials: Physicochemical Characterizations and Pharmacokinetic Evaluation of Pentazocine Solid Lipid Nanoparticles Against Inflammatory Pain Model

Zaheer Ullah Khan , Anam Razzaq , Ahsan Khan , Naeem Ur Rehman , Hira Khan , Taous Khan , Ashraf Ullah Khan , Farid Menaa Haroon Iqbal and Naveed Ullah Khan

Table S1. Independent variables predicted by DoE.

**Dependent Process Variables**	Particle size (nm)<br>Potential (mV)<br>PDI<br>Percentage Entrapment efficiency (EE%)<br>Percentage loading efficiency (LE%)	
**Independent process variables**	Levels	
	Minimum	Maximum
	-1	+1
Lipid	Stearic acid (S.A)	Cetyl alcohol (C.A)
Surfactant	Tween 80	Tween 20
Surfactant concentration (%)	2	3
Soya lecithin: lipid	2:7	1:8
Acetone: DCM	1:1	1:2
Homogenizer speed (rpm)	15000	16000
Homogenization time (min)	10	15

Table S2. PTZ drug release models at pH 1.2 and 6.8.

**Formulation**	**pH**	**R^2**			**Values of "n" for Korsmeyer-Peppas**
		Zero-order	1st -order	Higuchi - model	
**PTZ-Sol**	**1.2**	0.8781	0.8894	0.9424	0.527
	6.8	0.9851	0.9956	0.9893	0.650
**PTZ-SLNs**	1.2	0.8668	0.8790	0.9548	0.494
	6.8	0.9440	0.9673	0.9799	0.655

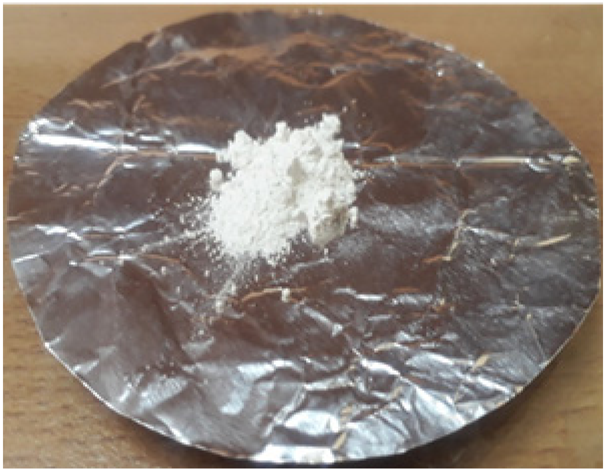

Figure S1. Fine powder of lyophilized SLN2.

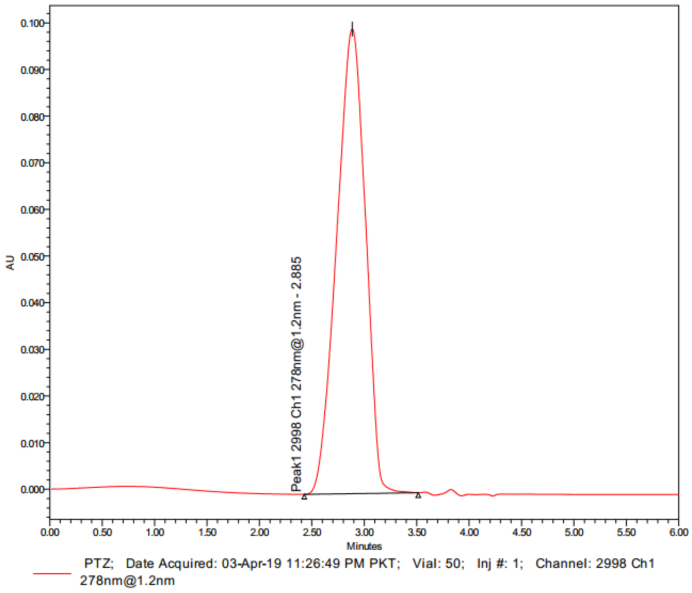

Figure S2. Chromatographic profile of free PTZ.

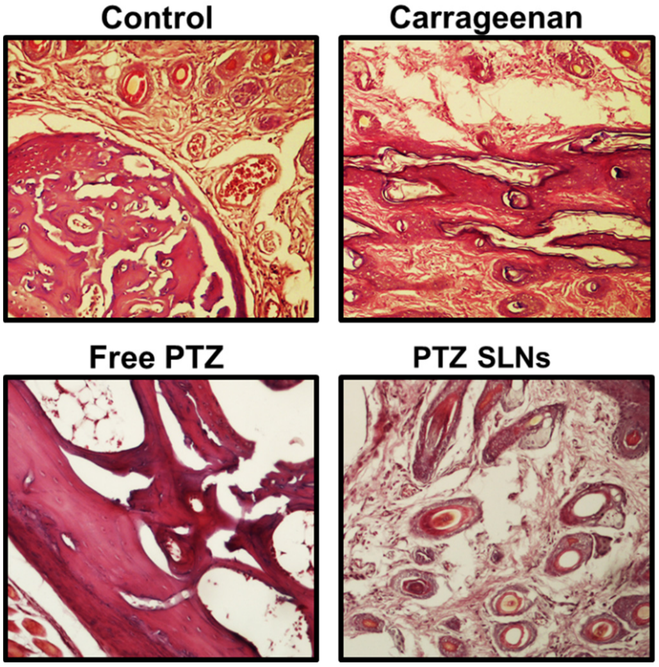

Figure S3. Effects of the PTZ-loaded SLN2 on the paw tissue architecture following carrageenan-induced inflammatory pain in Wistar rats.

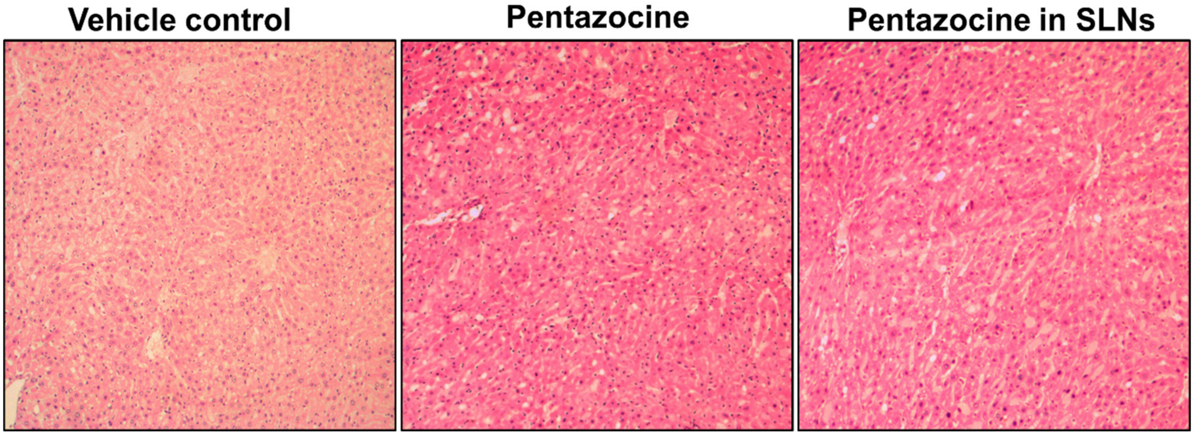

Figure S4. Histological effects of PTZ-loaded SLN2 on the liver of Wistar rats.
